# Engaging Communities in Emerging Infectious Disease Mitigation to Improve Public Health and Safety

**DOI:** 10.3201/eid3007.230932

**Published:** 2024-07

**Authors:** Michàlle E. Mor Barak, Shinyi Wu, Gil Luria, Leslie P. Schnyder, Ruotong Liu, Anthony Nguyen, Charles D. Kaplan

**Affiliations:** University of Southern California Marshall School of Business, Los Angeles, California, USA (M.E. Mor Barak);; University of Southern California Suzanne Dworak-Peck School of Social Work, Los Angeles (M.E. Mor Barak, S. Wu, L.P. Schnyder, R. Liu);; University of Southern California Viterbi School of Engineering, Los Angeles (S. Wu, A. Nguyen);; University of Haifa Faculty of Social Welfare and Health Sciences, Haifa, Israel (G. Luria, C.D. Kaplan)

**Keywords:** pandemic preparedness, public health policies, health behaviors, information sources, health-safety climate theory, COVID-19, community health, global health, bioterrorism and preparedness

## Abstract

The COVID-19 pandemic highlighted the need for potent community-based tools to improve preparedness. We developed a community health-safety climate (HSC) measure to assess readiness to adopt health behaviors during a pandemic. We conducted a mixed-methods study incorporating qualitative methods (e.g., focus groups) to generate items for the measure and quantitative data from a February 2021 national survey to test reliability, multilevel construct, and predictive and nomologic validities. The 20-item HSC measure is unidimensional (Cronbach α = 0.87). All communities had strong health-safety climates but with significant differences between communities (F = 10.65; p<0.001), and HSC levels predicted readiness to adopt health-safety behaviors. HSC strength moderated relationships between HSC level and behavioral indicators; higher climate homogeneity demonstrated stronger correlations. The HSC measure can predict community readiness to adopt health-safety behaviors in communities to inform interventions before diseases spread, providing a valuable tool for public health authorities and policymakers during a pandemic.

The COVID-19 pandemic demonstrated the influence community attitudes have on the health behaviors of its residents (e.g., wearing facemasks, accepting vaccines). Evidence on COVID-19 spread indicates that within-community contexts affect individual perceptions regarding taking precautions and following government guidelines and policies vary (*1*–*3*). Because COVID-19 and other infectious diseases spread mainly among persons sharing a physical environment, behaviors of members of a geographic community can influence transmission. 

Climate in the context of health-safety refers to shared perceptions within a community about the importance of maintaining and supporting behaviors that protect residents from infectious diseases. Climate theory postulates that individual persons perceive how others in their group expect them to behave and shape their behaviors accordingly (*4*). From a social psychology perspective, climate refers to the shared perceptions of members of an organization, group, or community concerning the procedures, practices, and kinds of behaviors that are rewarded and supported within the unit (*5*,*6*). Tests of climate theory related to community characteristics (e.g., service, productivity, innovation, inclusion) have demonstrated climate to be an effective predictor of behaviors (*7*,*8*). Previous research suggests that tools to measure community climate can help identify differences in levels of health-safety climate (HSC level) among communities and extent of agreement among residents within a community (climate strength) (*5*,*9*). 

We defined communities as persons living in the same geographic area, having substantial interactive relations, and holding many attitudes in common (*10*). Data from the COVID-19 pandemic emphasize the central role of community dynamics in viral transmission (*11*,*12*). Community-based research demonstrates that interactions between persons within geographic communities enable both transmission of infectious diseases among residents and emergence of shared perceptions, which can be used in efforts to prevent and control spread of the disease. Studies have confirmed that shared norms, values, and behaviors exist in communities and influence the perceptions and behaviors of community members (*13*–*15*). However, other studies indicate that perceptions about the climate within a group may vary among individual persons (*7*,*9*). 

Mischel’s theoretical formulation of strong situations (i.e., communities with high HSC strength) explains the possible effects of climate strength (*16*). Degree of ambiguity about appropriate health-safety behaviors within communities differs on the basis of climate strength; strong situations demonstrate little ambiguity and weak situations greater ambiguity among members about perceptions and expectations regarding appropriate behavior. In strong-climate communities, individual responses vary little, whereas in weak-climate communities, variability is greater (*16*–*18*). Thus, climate strength may act as a moderator between climate level and behavioral outcomes. Members of communities with stronger climate levels will achieve greater consensus about the perceived necessity of community-related climate interventions (e.g., health-safety behaviors during a pandemic). Groups with greater consensus among members more consistently perceive the need to adopt health-safety behaviors, resulting in a stronger relationship between climate level and behaviors. 

Community health-safety behaviors are critical for preventing transmission of infectious diseases and returning to normal life through mitigating disease spread depends heavily on community behaviors. Having a tool to measure community HSC is essential for informing decisions and strategies to limit the spread of disease and combat variants during a pandemic. Some efforts to develop that measure have been undertaken at the organizational level, but no measure has been available at the community level (*19*,*20*). To address this gap, we developed a measure of the strength of community climate levels for adopting health-safety behaviors. During phase I, we used qualitative data derived from community-based focus groups, and during phase II, we validated the measure with data from a nationally representative sample. To assess reliability, we hypothesized that the HSC measure would demonstrate high internal consistency. To assess multilevel construct validity (*21*,*22*), we hypothesized that a sufficient level of agreement in HSC perceptions will exist among members of geographic communities. To assess predictive validity (*23*,*24*), we hypothesized that HSC level will be positively related to health-safety behaviors. To assess nomologic validity (*25*) (Figure 1), we hypothesized that HSC strength will moderate the relationship between HSC levels and health behaviors. The full research protocol was approved by the institutional review board at the University of Southern California (Los Angeles, CA, USA).

**Figure 1:**
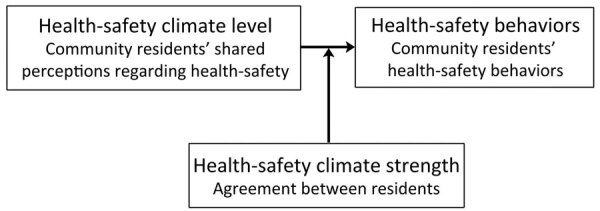
Diagram showing moderating effect of relationship health-safety climate strength on health-safety climate level leading to health-safety behaviors.

## Methods 

### Phase I—Generating the HSC Measure

#### Sample and Procedure

We conducted a series of focus groups from 4 diverse communities, each predominantly Asian, Latinx, Black, and White, across a large urban county in California. The groups resided in the same geographic area and had similar proportions of racial/ethnic composition; we tried to represent participants proportionally by age, sex, and socioeconomic status. With the help of community gatekeepers, social media, and messaging applications, we used purposive and snowball sampling to select participants and reduce bias. Ten focus groups (n = 39 persons) were organized; 12 (30.77%) participants were Latinx, 11 (28.21%) Chinese/Taiwanese, 9 (23.08%) White, and 7 (17.95%) Black. By age group, 17 (43.59%) participants were 18–39 years, 14 (35.90%) 40–59 years, and 8 (20.51%) 60–79 years. Most (30; 76.92%) participants were women, 23 (58.97%) had children, 25 (64.1%) had a college degree, and 24 (61.54%) reported a religious affiliation (Protestant, Catholic, Buddhist, or Jewish). 

Using climate theory as a theoretical background, we brainstormed potential topics for inclusion in the focus groups. Questions focused on participants’ perceptions and observations of pandemic-related behaviors among the residents in their communities. Each focus group session lasted ≈75 minutes, was hosted on Zoom, and was video recorded. 

#### Data Analysis 

We used a 4-step approach to identify items for inclusion in the HSC measure (*26*). In step 1, focus group transcripts were generated automatically from the video recordings. One team member reviewed the transcripts for accuracy by comparing them to the recordings, and another member completed an additional review. All personally identifiable information was removed. All transcripts were uploaded into NVivo 12 (https://help-nv.qsrinternational.com/12/win/v12.1.115-d3ea61/Content/welcome.htm) for analysis. We used deductive thematic analysis during the initial coding process to generate a provisional codebook and coding framework (*27*,*28*). We used the codebook to enhance the accuracy and consistency of coding, as well as navigate the pragmatic demands of research (*28*). Examples of coded elements included behavioral expectations (e.g., behavioral cues), COVID-19–related policy (e.g., adherence, enforcement), and reasons persons do or do not follow guidelines. Four team members were trained as coders, and each focus group transcript was analyzed independently by 2 coders. The coders met in analytic seminars to discuss the coding hierarchical process and modify the codebook of parent (main) codes and related child (minor) subcodes. New parent and child codes emerged during data analysis, and we added them to the codebook. Finally, the coders identified overarching themes representative of core categories and storylines from the qualitative data. 

In step 2, we used a top-down/bottom-up approach to guide generation of measure items (*6*). Items were statements about the measure that respondents were asked to endorse. These statements were validated and used to construct the final scale. Initial themes identified in step 1 guided further brainstorming of sample items representative of the final general themes. Examples for each item generated were drawn directly from focus group discussions to represent variation in context and topic. For example, 1 item generated was the statement “In my community, members are likely to tell someone to follow COVID health-safety guidelines (wearing masks, social distancing, vaccinations, etc.).” Another was “In my community, members make others feel uncomfortable (make fun of, sarcastic remarks, etc.) for following COVID health-safety guidelines (wearing masks, social distancing, vaccinations, etc.).” Next, we used a bottom-up approach to review examples related to the themes developed in step 1. We then reviewed each example to assess whether we needed to develop a new item. This process generated 62 items; we analyzed those items for overlap and removed duplicates. 

In step 3, we developed checklist statements of items that unambiguously identified behaviors related to COVID-19 health-safety. We first rank-ordered checklist statements on the basis of our experience collecting and analyzing focus group data. Using the same checklist, we rank-ordered items on the basis of perceived relative importance. We discussed the adequacy of external referents and refined the wording of the final set of statements to ensure relevance and minimize response acquiescence bias. This process continued until we reached consensus. 

In step 4, we finalized the HSC measure using Q-sort analysis by first clustering similar statements into piles and assigning names to each pile. This analysis provided a key for interpreting the underlying measurement construct and enhancing reliability of clustering. We then revised and finalized the measure for field testing (*29*). 

### Phase II—Validating the Measure 

#### Sample and Procedure 

Using qualitative data obtained from the focus groups in phase I, we developed a 20-item HSC measure. To validate the measure, we incorporated the items into the Understanding Coronavirus in America tracking survey, which is an expansion of the Understanding America Study (UAS) (*30*) that includes questions related to the COVID-19 pandemic. The UAS is an ongoing internet panel of ≈9,500 adult respondents who represent households across the United States. Beginning in 2020 and continuing throughout the pandemic, UAS sent out waves of the tracking surveys to inquire about personal behaviors and perceptions relevant to COVID-19.

We selected questions from the Understanding Coronavirus in America tracking survey relevant to behaviors during the COVID-19 pandemic to serve as a standard for the predictive validity of the HSC measure. Questions included: “Which of the following have you done in the last seven days to keep yourself safe from coronavirus: 1) Avoid public spaces, gatherings, or crowds; 2) Avoid contact with high-risk persons; 3) Avoid eating at restaurants; 4) Wear a face mask or some other face coverings; 5) Remain in your residence except for essential activities or exercise; and 6) Wash hands with soap or used hand sanitizer several times a day.” Potential responses for each question were yes, no, or unsure. 

To enable reporting of the HSC measure in community subgroups, we selected all ZIP (postal) codes within the UAS that had ≥10 respondents, resulting in 153 postal codes. In February 2021, we distributed English- and Spanish-language versions of the HSC measure to all UAS participants residing in one of the 153 postal codes (n = 2,359). We received responses from 1,672 (70.9%) participants. We excluded from analysis 24 (1.4%) respondents with missing data (final n = 1,648). 

To protect the identities of respondents and ensure a sufficient number of respondents from each geographic community, we created a community for each postal code, identified by the first 3 digits of the postal code. To mask respondent identifiers, the UAS survey team created a variable that provided respondents with an index number associated with their 3-digit postal-code community. Only postal-code communities with ≥10 respondents were included in the analyses. The final sample included 1,438 respondents who were nested in 49 communities. The average number of respondents from each community was 29.34 (range 10–319). 

#### Data Analysis

We used an exploratory factor analysis (EFA) of the curated UAS data to determine construct validity. The EFA used principal components analysis with varimax rotation to uncover underlying factor structures for each of the 20 HSC items using data from the 1,438 respondents. We performed 2 tests for the fit of factor analysis to the data: Bartlett’s test of sphericity, which yielded a significant test statistic of 8,961.302 (p<0.001), and the Kaiser-Meyer-Olim test, which yielded a test statistic of 0.924. Both tests indicated that factor analyses were appropriate. We calculated Cronbach α for the 20 identified items to determine internal consistency of the measure. To assess multilevel construct validity (*21*,*22*), we calculated an index for HSC strength at the community level using group-level interrater agreement measured by the ratio of within-group variance (r_WG_) (*31*), which is the proportion of observed group variance relative to expected random variance (*32*). r_WG_ ≥0.70 indicates satisfactory group-level agreement (*31*). 

We assessed predictive validity using a series of multilevel logistic regression analyses among the HSC measure and behavioral indicators while controlling for age and sex (*23*,*24*). We used behavioral indicators from the Understanding Coronavirus in America survey (*30*) and included avoiding public places and wearing a face mask. The dependent variable was whether the respondent had performed each behavioral indicator (yes = 1, no = 0). Because the number of unsure responses was relatively small (n = 8 for washing hands to n = 21 for avoiding public places), we excluded those responses from the analyses. Independent variables included the HSC measure, age, and sex (male = 0, female = 1). 

We assessed nomologic validity using multilevel logistic regression analyses (*25*). We explored the moderating effects of climate strength on the relationship between HSC level and the same UAS behavioral indicators used in the predictive validity procedure. We selected behavioral indicators from Centers for Disease Control and Prevention recommendations: avoiding public places, avoiding contact with high-risk persons, avoiding eating at restaurants, washing hands, wearing a face mask, and remaining in one’s own residence except for essential activities, including exercise. We performed multilevel modeling analyses with r_WG_ as a moderator between HSC and behavioral outcomes, controlling for age and sex; persons were nested within groups at the community level. We measured HSC and health-safety behaviors at the individual level and climate strength at the community level. 

## Results

### Phase I

We generated a 20-item community HSC measure that included questions related to following health-safety behaviors, including community mask wearing, distancing guidelines, and the effects of community leadership, among others (Appendix). The HSC measure incorporated a 5-point Likert scale with responses ranging from 1 (strongly disagree) to 5 (strongly agree). To avoid response-set bias, we applied reverse wording to negative statements, when appropriate. For example, “In my community, members will socially distance themselves from someone who is not wearing a mask (keep away from someone)” and “In my community, persons believe that their freedom to decide what is right for them is more important than following COVID health-safety guidelines (wearing masks, social distancing, vaccinations, etc).” The HSC measure score was determined quantitatively by totaling Likert scale responses from each item and dividing by 20. Because missing data were minimal (n = 14, 0.8%), we performed factor analysis only when respondents answered all 20 questions. HSC measure scores ranged from 1.3 to 4.7; higher scores indicated higher community HSC levels. 

### Phase II

#### Community HSC Level

Based on the scree plot and factor loadings, 2 factors accounted for most of the variance in the items proposed to measure community HSC: factor 1 had an eigenvalue of 6.38 and factor 2 a value of 2.05, explaining 42.12% of the variance (factor 1 = 31.88%, factor 2 = 10.24%). Review of the item content and their loadings indicated that all positive items loaded on factor 1, whereas all negative (reverse-coded) items loaded on factor 2. Because we could determine no other explanation for the formation or potential reversed-item biases, we concluded the measure was unidimensional (*33*). All 20 items were included in the final measure (reverse-coding the negative statements). A Cronbach α of 0.87 for the overall measure indicated strong internal consistency. 

#### Community HSC Agreement Strength

High agreement was demonstrated in climate perception (strength of climate) levels (median r_WG_ = 0.9). All communities sampled in the study had strong climates (r_WG_ >0.73). Analysis of variance revealed significant differences among community climate levels (F = 10.65; p<0.001). Thus, members of a given community agreed on the climate level within their community, but communities had significantly different climate levels between one another.

#### HSC Relationships with Behavioral Indicators

Multilevel modeling analyses between the HSC measure and COVID-19 behavioral indicators demonstrated that the higher the HSC level, the more likely persons within the group were to avoid public places (b = 0.73; p<0.001), avoid contact with high-risk persons (b = 0.87; p<0.001), avoid eating at restaurants (b = 0.52; p<0.001), wash their hands frequently (b = 0.68; p<0.01), wear a face mask (b = 1.25; p<0.001), and remain in their own residence except for essential activities or to exercise (b = 0.66; p<0.001) (Table).

**Table:** Multilevel logistic regression analysis of health-safety climate and behavioral outcomes, controlling for age and sex, as part of developing a community HSC measure to assess readiness to adopt health behaviors during a pandemic*

Category	Avoid public spaces, n = 1,048	Avoid contact with high-risk persons, n = 992	Avoid eating at restaurants, n = 1,048	Wash hands, n = 1,060	Wear a face mask, n = 1,061	Remain in residence except for essential activities, n = 1,064
Predictor variables						
HSC level†	0.73‡	0.87‡	0.52‡	0.68§	1.25‡	0.66‡
Age¶	0.01	0.02†	0.01	0.01	–0.01	0.02‡
Female sex#	0.27**	0.42**	0.04	0.82‡	0.15	0.24
Moderation analysis with HSC strength as moderator						
HSC level‡	–9.03**	–12.59§	–6.83	–17.74**	–1.42	–4.18
HSC strength††	–28.19**	–42.86§	–19.63	62.56**	–5.02	–11.71
HSC level × HSC strength	11.12§	15.37§	8.33	20.97**	3.01	5.49
Age¶	0.01	0.02‡	0.01	0.01	–0.01	0.02‡
Female sex#	0.25	0.039**	0.04	0.79§	0.17	0.24

*Five records were excluded from each regression model because of missing information. HSC, health-safety climate. †HSC level ranged from 1.3 to 4.7 (mean 3.17, SD 0.49) ‡Statistical significance at p<0.001 level based on the Wald test. §Statistical significance at p<0.01 level based on the Wald test. ¶Age ranged from 19 to 111 y (mean 47.42 y, SD 16.23 y). #Sex: 890 participants (62.46%) were female and 535 (37.54%) were male. **Statistical significance at p<0.05 level based on the Wald test. ††HSC strength (r_WG_) ranged from 0.69 to 0.95 (mean 0.88, SD 0.03).

#### HSC as a Moderator

**Figure 2:**
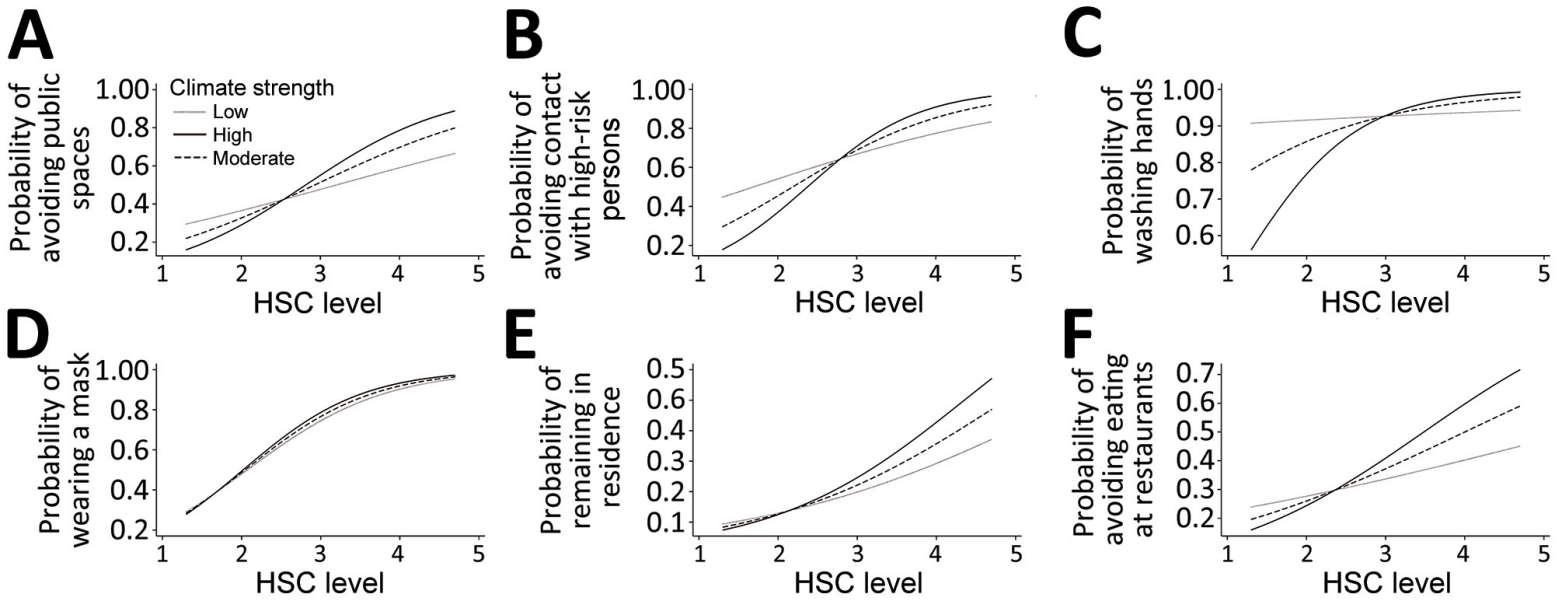
HSC and behavioral outcomes by HSC strength. Moderate HSC is represented by the mean of r_WG_; low HSC is represented by 1 SD below and high by 1 SD above the mean of r_WG_. HSC, health-safety climate.

Multilevel modeling analyses with r_WG_ as moderator demonstrated that HSC strength moderated the relationship between HSC level and behavioral indicators, including avoiding public spaces (b = 11.12; p<0.01), avoiding contact with high-risk persons (b = 15.37; p<0.01), and washing hands frequently (b = 20.97; p<0.05) (Table; Figure 2). Moderation effects were not found for the other 3 indicators: avoiding restaurants, wearing a face mask, and remaining in own residence. Communities with higher climate homogeneity demonstrated a stronger correlation between HSC and behavioral outcomes than groups with lower climate homogeneity. 

## Discussion

We generated and validated an HSC measure as a tool to measure community climate level for health-safety. Using qualitative data from 4 distinct geographic communities, we generated 20 items to assess the level of community climate for health-safety behaviors. To validate the measure, we tested 4 hypotheses using nationally representative samples from the UAS. The HSC measure demonstrated high internal consistency. We found strong agreement regarding HSC perceptions among members within single geographic communities and demonstrated that HSC level was positively related to health-safety behaviors. Finally, we demonstrated that HSC strength moderates the relationship between climate level and health behaviors. The multilevel modeling analyses supported HSC strength as a moderator between HSC level and behavioral indicators. Communities with higher climate homogeneity showed stronger correlation between HSC and behavioral outcomes. Overall, the HSC measure demonstrated internal consistency and strong predictive, multilevel, and nomologic validities. Of note, the HSC measure can be applied to a variety of infectious diseases and used in public health studies of current and future epidemics and pandemics. 

Consistent with climate theory, the HSC measure clearly demonstrated that community HSC can predict behaviors in a variety of communities (*5*,*6*). Our study focused on persons who live in the same geographic area; members of geographically close communities tend to have substantive interactive relations and common attitudes, both of which are relevant to the transmission levels of infectious diseases (*10*). That dynamic was evident during the COVID-19 pandemic, in which infection rates differed between neighborhoods (*11*,*12*). 

Our findings might clarify the general effect of normative social influence on decision-making, including community climate regarding informal health-safety mores and norms and formalized rules and procedures. Understanding underlying perceptions of social and health-related behaviors of community members is vital for mitigating disease spread during a pandemic (*34*), especially before vaccine availability and as variants emerge. During the COVID-19 pandemic, many communities over time exhibited reduced tolerance and growing resentment toward external controls and enforcement of behavioral guidelines by authorities (*35*). Given the diminishing returns of such formal enforcements over time, enlisting local communities and their leaders to create climates that encourage residents to voluntarily take health-safety actions is critical. Furthermore, the HSC measure can be especially useful given the heterodoxy of scientific opinions regarding the most effective mitigation strategies, guidelines, and policies at the community level (*3*). The HSC measure can also help democratize policymaking in public health pandemic control by systematically accounting for and addressing perspectives among community members. 

Further analysis at the community level is needed. Of particular interest are studies examining the interrelationships of community HSC and health-safety decision-making during a pandemic with regard to competing social values, such as following government guidelines versus rights of individual determination. Similarly, differences in individual perceptions regarding the risk of infection, perceived risks in family and friend circles, and risks to the public should be assessed. Such assessments could inform future research on how trust of particular sources for providing information and personal preferences affect health-safety behaviors. Finally, future research should measure the relationship between community HSC and objective data (e.g., infection rates, number of persons hospitalized because of infection). 

Among our study’s strengths, we combined an in-depth qualitative process to generate measurable items with data from diverse communities and use of a large national cohort for validation. Among limitations, we performed our analysis at the community level, but because of the nature of the large national sample used for validation and the need to protect participants’ anonymity, the best proxy available was to designate communities based on postal codes. Analyses of smaller geographic communities may provide more accurate results; thus, future studies should sample smaller communities (e.g., neighborhoods). Although major racial/ethnic groups were represented in our focus groups, groups were not proportional with respect to sex, age, and family size. Because of countywide research regulations during the COVID-19 pandemic, we were unable to recruit participants in person or conduct face-to-face focus groups. Future research in this area should include more representative samples for recruiting focus groups to generate measurable items. Finally, reliance on self-reports from respondents may have introduced single-source bias. 

In conclusion, during pandemics, shared perceptions within communities and the consequent behaviors of community members can affect transmission of infectious diseases (*13*–*15*). Building on climate research, our HSC measure can assist in assessing HSC level and strength in different communities and identifying communities in which risk for infection spread is high. The HSC measure can assist public health authorities and policymakers in preventing and controlling the spread of a pandemic by enabling focused interventions to improve health-safety climates before disease spread occurs or is amplified. Thus, the HSC measure can serve as a valuable tool for pandemic preparedness and response as well as for public health studies of current and future pandemics. 

Appendix List of items generated by Phase I focus groups to develop the Community Health-Safety Climate Measure
