# Supplementary material for: Engaging Communities in Emerging Infectious Disease Mitigation to Improve Public Health and Safety
Source: Emerg Infect Dis. 2024 Jul;30(7):1390–7. doi: 10.3201/eid3007.230932 (PMC11210660; doi:10.3201/eid3007.230932)
Supplement: Appendix — List of items generated by Phase I focus groups to develop the Community Health-Safety Climate Measure [file 23-0932-Techapp-s1.pdf]

Article DOI: https://doi.org/10.3201/eid3007.230932

EID cannot ensure accessibility for supplementary materials supplied by authors. Readers who have difficulty accessing supplementary content should contact the authors for assistance.

Engaging Communities in Emerging Infectious Disease Mitigation to Improve Public Health and Safety

Appendix

The Community Health-Safety Climate (HSC) Measure

1. In my community, members are likely to tell someone to follow COVID health-safety guidelines (wearing masks, social distancing, vaccinations, etc.).

2. In my community, people believe that their freedom to decide what is right for them is more important than following COVID health-safety guidelines (wearing masks, social distancing, vaccinations, etc.).

3. In my community, members make others feel uncomfortable (make fun of, sarcastic remarks, etc.) for following COVID health-safety guidelines (wearing masks, social distancing, vaccinations, etc.).

4. In my community, members will socially distance themselves from someone who is not wearing a mask (keep away from someone).

5. In my community, leaders (for example, elected officials, police officers, doctors, and clergy) do not follow COVID health-safety guidelines (wearing masks, social distancing, vaccinations, etc.).

6. My community has visible COVID health-safety postings indicating expected health-safety behaviors (wearing masks, social distancing, vaccinations, etc.).

7. Many people in my community believe that COVID is not really a dangerous disease.

8. People in my community expect others to take the vaccine as soon as it is available to them.

9. In my community, members expect others to follow COVID health-safety guidelines (wearing masks, social distancing, getting vaccinated, wearing masks, etc.).

10. People in my community avoid shopping and entertainment places that are strict in enforcing COVID-safety behaviors.

11. People in my community expect those who are COVID positive to quarantine (stay home and not interact with others) for 10 days, and those who have been exposed to COVID to stay home and not interact with others following CDC-recommended isolation or quarantine period.

12. People in my community feel it is not necessary to follow COVID health-safety guidelines (wearing masks, social distancing, vaccinations, etc.) around people they trust, such as family, friends, and close colleagues.

13. Most people in my community have replaced physical greetings (handshakes, hugs) with other forms, such as elbow bumps, fist bumps, and waves.

14. In my community, if someone reminds others to follow COVID health-safety guidelines (wearing masks, social distancing, vaccinations, etc.), they encounter refusal, conflict, or resentment.

15. In my community, members expect others to have only small gatherings of family and friends in their home.

16. In my community, holidays and social events are celebrated in a COVID-safe way (wearing masks, social distancing, requiring vaccinations, etc.).

17. In my community, businesses and entertainment places (such as gyms, sporting arenas, religious institutions, etc.) do not require nor check if you are vaccinated (for indoor spaces).

18. In my community, businesses and entertainment places enforce COVID health-safety guidelines (wearing masks, social distancing, requiring vaccinations, etc.) for customers and visitors.

19. Members of my community prefer going to businesses (doctors'/veterinarians' offices, hairdressers, etc.) that enforce COVID health-safety guidelines (wearing masks, social distancing, requiring vaccinations, etc.).

20. In my community, people are critical of leaders/elected officials who do not always follow COVID health-safety guidelines (wearing masks, social distancing, vaccinations, etc.).
